# Artificial two-dimensional polar metal at room temperature

**DOI:** 10.1038/s41467-018-03964-9

**Published:** 2018-04-18

**Authors:** Yanwei Cao, Zhen Wang, Se Young Park, Yakun Yuan, Xiaoran Liu, Sergey M. Nikitin, Hirofumi Akamatsu, M. Kareev, S. Middey, D. Meyers, P. Thompson, P. J. Ryan, Padraic Shafer, A. N’Diaye, E. Arenholz, Venkatraman Gopalan, Yimei Zhu, Karin M. Rabe, J. Chakhalian

**Affiliations:** 1 Department of Physics and Astronomy, Rutgers University, Piscataway, NJ 08854 USA; 2 CAS Key Laboratory of Magnetic Materials and Devices, Ningbo Institute of Materials Technology and Engineering, Chinese Academy of Sciences, Zhejiang 315201 Ningbo, China; 3 Department of Physics and Astronomy, Louisiana State University, Baton Rouge, LA 70803 USA; 4 Department of Condensed Matter Physics and Materials Science, Brookhaven National Laboratory, Upton, NY 11973 USA; 5 Department of Physics, University of California Berkeley, Berkeley, CA 94720 USA; 6 Department of Materials Science and Engineering and Materials Research Institute, Pennsylvania State University, University Park, PA 16802 USA; 7 Department of Physics, University of Arkansas, Fayetteville, AR 72701 USA; 8 Department of Physics, Indian Institute of Science, Bangalore, 560012 India; 9 XMas CRG, European Synchrotron Radiation Facility, Cedex 38043 Grenoble, France; 10 Advanced Photon Source, Argonne National Laboratory, Argonne, IL 60439 USA; 11 School of Physical Sciences, Dublin City University, Dublin, 9 Ireland; 12 Advanced Light Source, Lawrence Berkeley National Laboratory, Berkeley, CA 94720 USA

## Abstract

Polar metals, commonly defined by the coexistence of polar crystal structure and metallicity, are thought to be scarce because the long-range electrostatic fields favoring the polar structure are expected to be fully screened by the conduction electrons of a metal. Moreover, reducing from three to two dimensions, it remains an open question whether a polar metal can exist. Here we report on the realization of a room temperature two-dimensional polar metal of the B-site type in tri-color (tri-layer) superlattices BaTiO_3_/SrTiO_3_/LaTiO_3_. A combination of atomic resolution scanning transmission electron microscopy with electron energy-loss spectroscopy, optical second harmonic generation, electrical transport, and first-principles calculations have revealed the microscopic mechanisms of periodic electric polarization, charge distribution, and orbital symmetry. Our results provide a route to creating all-oxide artificial non-centrosymmetric quasi-two-dimensional metals with exotic quantum states including coexisting ferroelectric, ferromagnetic, and superconducting phases.

## Abstract (web summary)

Materials that combine metallic behaviour with stable electric polarization are scarce despite being proposed in the 1960s. Here the authors engineer a perovskite heterostructure where 2D polar metallic behavior coexists with built-in electric polarization from the displacement of B-site titanium cations.

## Introduction

In modern materials science, the design of new materials with emergent quantum ground states relies on the combination of structure–function and structure–composition relationships^1–11^. The emergence of polar metals^1–6^ is one of such non-trivial and counter-intuitive examples motivated by the search for unconventional pairing in non-centrosymmetric superconductors and topology-protected spin currents. Surprisingly, three extensive materials surveys^2,12,13^ have revealed that oxide compounds are particularly scarce as polar metals. Based on the type of atomic displacements, polar metals with perovskite structure fall into two main categories^2^—A-site (e.g., positional shifts of Li, Nd, and Ca ions in LiOsO_3_^3^, NdNiO_3_^4^, and CaTiO_3−*δ*_^2^, respectively) or B-site dominated (e.g., shift of Ti ions in BaTiO_3−*δ*_^2,5,6^) types. For the former category, recent theoretical work^2^ has suggested the absence of a fundamental incompatibility between the polarity and metallicity, whereas for the latter, polar displacements show a rapid decrease with increasing carrier concentration^2,5,6^. Moreover, since thin films naturally hold a great potential for applications and discovery of new physical phenomena^14^, creating polar metals via atomically thin layering is a promising approach. The challenge is that both free carrier screening and reduced dimensionality have been observed generally to suppress ferroelectric instabilities in perovskite oxides^15^.

In the following, we design tri-color titanate heterostructures made of a layered arrangement of the ferroelectric alkaline-earth titanate BaTiO_3_ (BTO), the paraelectric alkaline-earth titanate SrTiO_3_ (STO), and the Mott insulator rare-earth titanate LaTiO_3_ (LTO). In this design, there are two inequivalent interfaces, BTO/STO and STO/LTO. The design idea is to transfer electrons from LTO into the STO layers forming two-dimensional electron gas (2DEG) at the interfaces^16,17^, which have a shared polar structure due to the presence of ferroelectric BTO^18–21^. In this paper, we present experimental measurements and first-principles calculations to show that a 2D polar metal is thus realized in this tri-color structure with coexisting polar displacements of Ti and O sublattices as well as metallicity in TiO_2_ atomic layers at the 2DEG interfaces.

## Results

### Two-dimensional electron gas

**Fig. 1:**
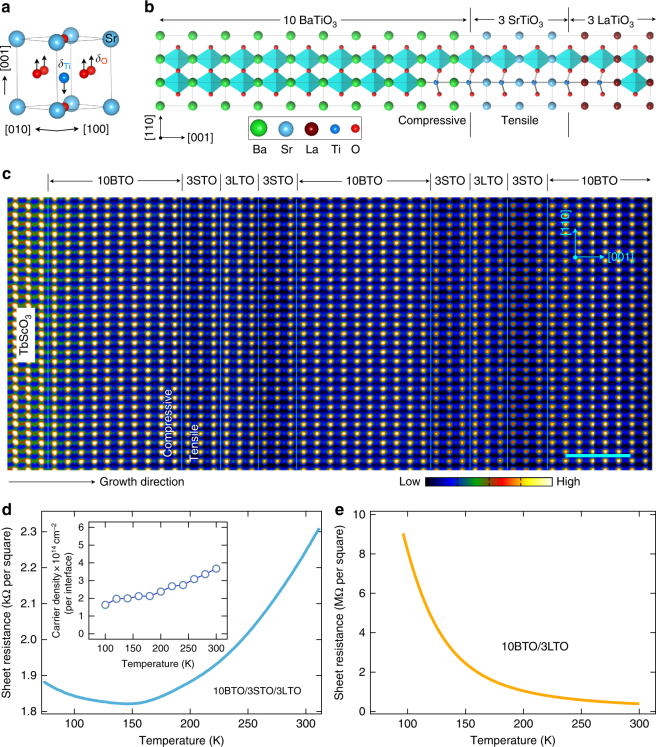
Design and synthesis of BTO/STO/LTO superlattices. **a** Schematic polar displacements of Ti cations (*δ*_Ti_, down arrow) and O anions (*δ*_O_, up arrows) relative to the centrosymmetric plane of the corresponding unit cell. **b** Sketch of tri-color heterostructure for designer two-dimensional polar metal. Notably, the STO layer is under tensile strain (~1.3%), whereas the BTO layer is under compressive strain (~−1.1%). **c** HAADF-STEM image of 10BTO/3STO/3LTO on TbScO_3_ substrate taken along [110] direction. The cyan scale bar is 2 nm. **d** Temperature-dependent sheet resistance (green curve) and carrier density (inset) of 10BTO/3STO/3LTO demonstrating a metallic behavior with large carrier density. **e** Temperature-dependent sheet resistance of 10BTO/3LTO showing an insulating behavior

Figure 1 shows the emergence of 2DEG at the STO/LTO interfaces of BTO/STO/LTO. Experimentally, ultra-thin tri-color titanate superlattices consisting of (BTO)_10_/(STO)_3_/(LTO)_3_ (where the subscript refers to the number of unit cells) as well as reference samples of (BTO)_10_/(LTO)_3_ superlattice and BTO thin film were synthesized on TbScO_3_ (110) single-crystal substrates by pulsed layer deposition in a layer-by-layer mode (see Fig. 1a, b, Supplementary Fig. 1, and Methods for more details). High crystallinity of the layers and good epitaxy were confirmed by in situ reflection-high-energy electron diffraction (RHEED) (Supplementary Fig. 1). To determine the atomic-scale structure of the samples, the interfacial structure and composition of BTO/STO/LTO were investigated by cross-sectional scanning transmission electron microscopy (STEM) with electron energy-loss spectroscopy (EELS). Figure 1c shows a high-angle annular dark-field (HAADF) STEM image of the tri-color superlattice, revealing high-quality continuous and coherent interfaces without phase separation. In the *Z*-contrast HAADF image, the expected layer thickness and designed sequence of three layers—…LTO/STO/BTO/STO/LTO…—are clearly distinguishable by the different intensities due to the difference in scattering power of the layers. Additionally, as shown in Supplementary Fig. 1d, the periodicity of the growth sequence was further confirmed by the periodic variation of the out-of-plane lattice parameters of individual BTO, STO, and LTO layers. In addition, to eliminate concerns about possible chemical inter-diffusion at the interfaces, EELS spectroscopic imaging was performed at the Sc L_2,3_-, Ti L_2,3_-, Ba M_4,5_-, and La M_4,5_-edges (Supplementary Fig. 2). As engineered with interfacial charge transfer, low-temperature electrical transport measurements of BTO/STO/LTO verified the expected metallicity and large carrier density of conduction electrons in all tri-color samples (*n*_c_ ~ 10^14^ cm^−2^, see Fig. 1d, e). In the metallic BTO/STO/LTO, due to insulating BTO/STO (3*d*^0^ band) and BTO/LTO (Fig. 1e) interfaces, the contribution of metallicity in BTO/STO/LTO is from the 2DEG of STO/LTO interfaces^16,17^. Notably, in sharp contrast to itinerant electrons at STO/LTO interfaces, the electrons at BTO/LTO interfaces are localized, the microscopic mechanism of which needs further study.

### Polar distortions of crystal structures

**Fig. 2:**
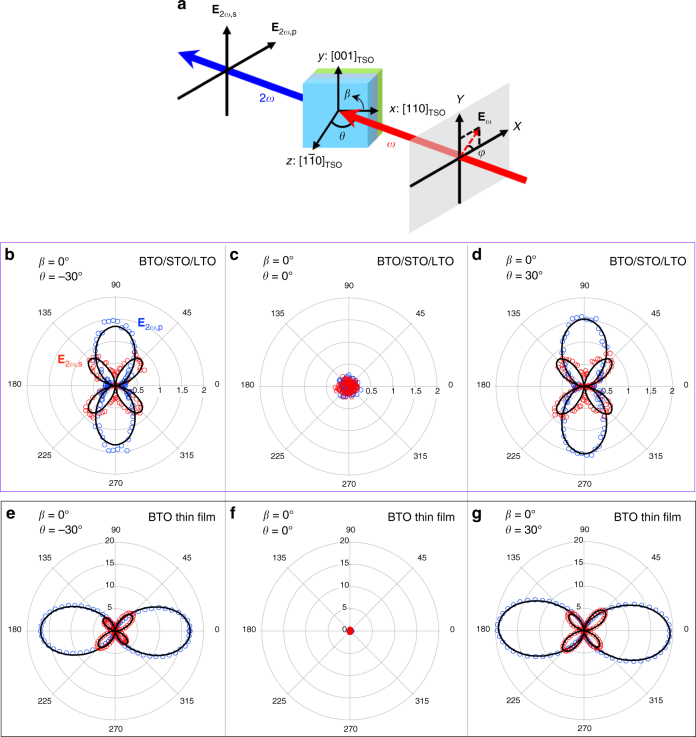
SHG polarimetry measurement. **a** Schematics of the far-field transmission SHG setup with crystallographic directions of TbScO_3_ substrate (subscript TSO) under sample coordinates (*x*, *y*, *z*). *E*_2*ω*,p_ (blue circles) and *E*_2*ω*,s_ (red circles) SHG signal on (**b**–**d**) BTO/STO/LTO and (**c**–**g**) BTO thin film (10 unit cells) are measured by rotating the incident polarization *φ* under three different incident angles *θ* = −30°, 0°, 30°. Data with *β* = 0° are plotted here (see Supplementary Fig. 3a–f for *β* = 90°). Theoretical modeling of SHG signal (black solid lines) indicates 4*mm* point group symmetry for BTO/STO/LTO with effective nonlinear optical coefficients *d*_33_/*d*_15_ ≈ −13.9, *d*_31_/*d*_15_ ≈ 1.3. In comparison, SHG signal on BTO thin film exhibits *mm*2 point group symmetry with *d*_33_/*d*_15_ ≈ 5.2, *d*_31_/*d*_15_ ≈ 0.1

To characterize the presence of polar displacements in BTO/STO/LTO, we carried out optical second harmonic generation (SHG) measurement, using a far-field transmission geometry as shown in Fig. 2a. Systematic polarimetry measurements on BTO/STO/LTO superlattice and BTO thin film were performed by measuring p- and s-polarized SHG signals *E*_2*ω*,p_ and *E*_2*ω*,s_ while rotating the incident polarization *φ* in two different orientations *β* = 0° and 90° for three incident angles *θ* = −30°, 0°, 30° (see Supplementary Fig. 3a–f for polarimetry with *β* = 90°). As seen in Fig. 2b–g, both BTO/STO/LTO superlattice and the BTO thin film show a clear SHG signal, indicating they are both polar structures. The decreased maximum SHG intensity in BTO/STO/LTO superlattice as compared to BTO thin film is consistent with the results shown further on that indicate opposite out-of-plane polarizations between several layers and sometimes within one layer of the superlattice, which will lead to a partial phase cancellation of the SHG response from different layers of the superlattice. In addition, remarkable differences in the SHG polarimetry were observed between BTO/STO/LTO superlattice and the BTO thin film. Specifically, the p-polarized SHG signal *E*_2*ω*__,__p_ of BTO/STO/LTO superlattice (Fig. 2b–d) has a maxima at incident polarizations of *φ* = 90° and 270°, whereas the BTO thin film (Fig. 2e–g) shows its maximum at incident polarizations of *φ* = 0° and 180°. A detailed theoretical modeling of SHG polarimetry revealed a net 4*mm* point group symmetry with an out-of-plane polarization for BTO/STO/LTO superlattice grown on orthorhombic TbScO_3_ (110) substrates, with effective nonlinear optical coefficients *d*_33_/*d*_15_ ≈ −13.9 and *d*_31_/*d*_15_ ≈ 1.3. In sharp contrast, the BTO thin film grown on the same substrate shows a net *mm*2 point group symmetry with an out-of-plane polarization, with *d*_33_/*d*_15_ ≈ 5.2 and *d*_31_/*d*_15_ ≈ 0.1. As a reference, we also performed SHG polarimetry on the *c*-domain of a bulk BaTiO_3_ single crystal (see Supplementary Fig. 3g–i), which indicates a 4*mm* symmetry and *d*_33_/*d*_15_ ≈ 3.3 and *d*_31_/*d*_15_ ≈ 0.3, which are close to the values obtained from the BTO thin film and very different from BTO/STO/LTO superlattice. The breaking of the fourfold to a twofold rotation symmetry in the 10 unit cells thick BTO thin film likely reflects the slight substrate anisotropy; this symmetry lowering is absent in the average 4*mm* symmetry of the thicker superlattice structure where this subtle substrate influence appears to have diminished. Ratios of nonlinear coefficients indicate intrinsic material properties as against microstructural effects; the dramatic changes in the ratios of between the BTO film and BTO/STO/LTO superlattice, strongly suggest that in addition to the BTO layers, the STO/LTO layers contribute a qualitatively distinct SHG behavior in the superlattice structure.

### Polar displacements at the atomic scale

**Fig. 3:**
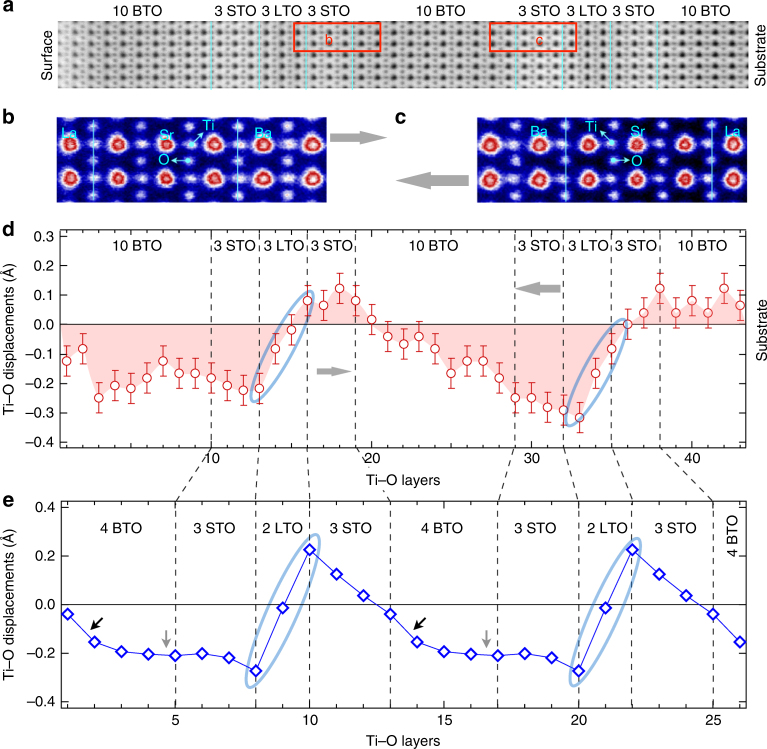
Polar displacements. **a** ABF-STEM image of 10BTO/3STO/3LTO along [110] direction. **b**, **c** Enlarged images of red rectangular areas in **a** showing atomic positions of Ba, Sr, La, Ti, and O ions across interfaces. Gray arrows (in **b**–**d**) indicate the reversal directions of Ti–O polar displacements. **d** Experimentally layer-resolved Ti–O polar displacements in a 10BTO/3STO/3LTO superlattice. Here the Ti–O polar displacements (or Δ_Ti–O_) are defined as oxygen displacement with respect to Ti along the out-of-plane. The error bar shows the standard deviations of the averaged measurements for each vertical atomic layer. **e** Theoretically layer-resolved Ti–O polar displacements in a 4BTO/3STO/2LTO superlattice. Black and gray arrows indicate negative and zero slopes of the Ti–O displacements, respectively, in BTO layers, whereas blue ellipses highlight positive slopes in LTO layers

Having established the presence of polar displacements averaged over the overall tri-color structure by SHG, next we investigate the microscopic details of centrosymmetry breaking of TiO_6_ octahedra in an atomic layer-resolved way. To address this, high-resolution HAADF- and ABF-STEM imaging were carried out, which allows to directly observe and extract the precise atomic positions of all constituent atoms, including oxygen across the interfaces. As shown in Fig. 3a–c, significant Ti–O polar displacements, i.e., relative shifts of Ti and O along the out-of-plane direction, are observed in the BTO/STO/LTO tri-color structure, which is consistent with the inversion symmetry breaking revealed by SHG. Additionally, to determine amplitudes and directions of the polar displacements, a detailed quantitative analysis of the ABF-STEM image (Fig. 3a) was performed. Figure 3d shows the evolution of Ti–O polar displacements layer by layer across the interfaces. The Ti–O polar displacements are found to be as large as 0.3 Å, that is almost 8% enlargement of the lattice parameters (~4 Å for bulk BTO, STO, and LTO). Moreover, these large Ti–O polar displacements not only exist in BTO but also extend deep into the STO and LTO layers. This behavior agrees well with SHG data and further corroborates the presence of the polarization in the STO layer. A striking feature is the periodic reversal of polar directions across the LTO layers. We attribute this to atomic displacements driven by local up–down symmetry breaking, typical of perovskite surfaces, at the STO/LTO interface. This non-switchable polar distortion propagates into the other layers: note, for instance, that the directions of polar displacements in the TiO_2_-atomic layers labeled by the gray arrows in Fig. 3b–d point in opposite ways. To understand the microscopic origin of the polar distortions observed in the STEM and SHG measurements, first-principles GGA + *U* calculation were performed. Figure 3e shows the calculated Ti–O polar displacements from ground-state atomic structure. The two dominant features, broken inversion symmetry from the net negative Ti–O displacements manifested in the BTO layers and the inversion of Ti–O polar displacements across LTO layers, are in a good agreement with the experimental data (Fig. 3d). The broken inversion symmetry is primarily driven by the polar distortion in BTO (~0.2 Å), and clearly absent in the symmetric STO/LTO superlattice (Supplementary Fig. 4). These polar distortions propagate deeper into STO layers, consistent with previous predictions for BTO/STO superlattices^18–20^. Near the STO/LTO interface, the Ti–O distortions are predominantly affected by the ionic screening of positively charged (LaO)^1+^ layers in which the Ti (O) ions move away (to) the center of LaO layers. This behavior results in the reversal of the Ti–O displacements across the LTO region^22^.

### Modulation of interfacial metallicity and polarity

**Fig. 4:**
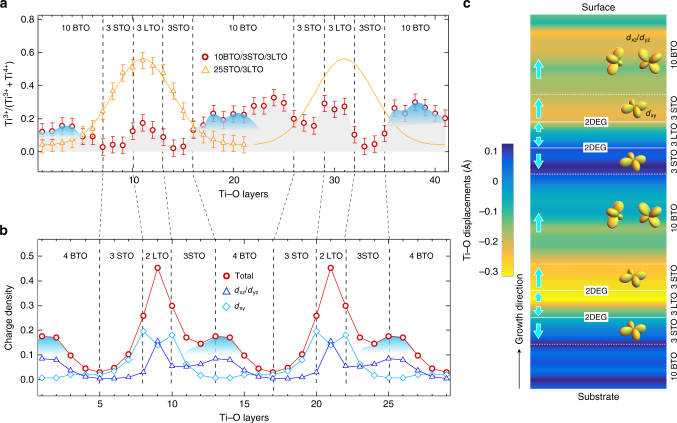
Modulation of electric polarization, charge distribution, and orbital symmetry. **a** Experimentally layer-resolved charge distribution of 10BTO/3STO/3LTO (red circles) and reference sample 25STO/3LTO (yellow triangles) measured by STEM/EELS. It is noted the yellow solid lines are guidelines for clarity. Here the error bar indicates the standard deviation between experimental and fitting spectra. Blue shadows (in** a**, **b**) indicate the anomalous charge distribution in BTO layers. **b** Calculated orbitally resolved charge distribution for each TiO_2_ layer in 4BTO/3STO/2LTO. Red circles indicate total projected density of states, whereas blue triangles and light-blue rhombuses label the contribution from *d*_xz_/*d*_yz_ and *d*_xy_ orbitals, respectively. **c** Illustration of coexisting electric polarization and two-dimensional electron gas with theoretically proposed in-plane *d*_xy_ state as the preferential occupation. The image is plotted based on experimental Ti–O polar displacements (Fig. 3d). White solid lines schematically indicate the positions of two-dimensional electron gases, whereas cyan arrows mark the direction switching of Ti–O displacements. The strength and direction of polarization are labeled by the color map from blue to yellow

Since the spin, charge and orbital degrees of freedom are intimately coupled in complex oxides containing transition metal ions with open shells, next we discuss the effect of the carrier modulation on the orbital structure of *d*-electrons. Experimentally, the interfacial distribution of charge was probed with atomic resolution STEM/EELS. In order to determine the valence state of Ti, we performed fittings of the Ti L_2,3_-edge EELS spectra across the interfaces by using reference spectra of Ti^4+^ from bulk BTO and STO and Ti^3+^ from bulk LTO. Figure 4a shows the distribution of Ti^3+^ (3*d*^1^) fraction by analyzing atomic layer-resolved EELS, to reveal a charge reconstruction across BTO/STO/LTO layers. As immediately seen, in contrast to conventional STO/LTO interfaces, where free carriers density decays exponentially with the distance from the interface^16,17^, our EELS data reveal the presence of an unusual charge modulation inside the BTO layers with the carrier density comparable or even higher than that of LTO layers (Fig. 4a). To understand the unusual charge accumulations observed in the BTO layers, we performed first-principles calculations. Figure 4b shows the atom-resolved orbital occupation for Ti-*d* states atom by atom along the *z*-direction defined parallel to the *c*-axis. The density of states in BTO layer exhibits a strong deviation from the exponential decay reported for STO/LTO hetetostructures^16,17,22^, showing consistent feature (blue shadows) with the experimental charge density distribution determined from EELS. More specifically, as seen in Fig. 4b, we find that around the LTO region, *d*_xy_ states are dominantly occupied and the occupation decays exponentially, whereas in the BTO region, *d*_xz_/*d*_yz_ states are mostly occupied with the density shifted toward to the left BTO/STO interface. The spatial separation of *d*_xy_ and *d*_xz_/*d*_yz_ states is the combined effect of the electrostatic energy and the crystal field splitting; namely, in the LTO region, the electrostatic potential from positively charged (LaO)^1+^ layers dominates and is screened by *d*_xy_ electrons having in-plane dispersions. In the BTO region, the out-of-plane (or apical) Ti–O distances becomes substantially larger than in-plane Ti–O distances due to the elongated *c*-lattice constant, which in turn lowers the on-site energy of *d*_xz_/*d*_yz_ orbitals and results in the large increase in the *d*_xz_/*d*_yz_ orbital occupancy compared to STO/LTO heterostructures^23^ (see also Supplementary Fig. 5). Around the STO/LTO interface, *d*_xy_ states are lower in energy relative to the *d*_xz_/*d*_yz_ states from the large modulation in the Ti to out-of-plane (apical) oxygen distance (+0.1/−0.15 Å)^22^ and vice versa for the BTO region due to the elongation of *c*-lattice constant increasing average Ti–O distance in the out-of-plane direction. The shift in the charge density can be understood from the polarization gradient of the Ti–O displacements. Around the BTO/STO interface on the left to the BTO region, the negative slope of the Ti–O displacements (black arrows in Fig. 3e) corresponds to the accumulation of the positive ionic charge, functioning as an attractive potential, whereas the slope on the right BTO/STO interface (gray arrows in Fig. 3e) is close to zero, resulting in the shift in the charge density to the left BTO/STO interface. In addition, the positive slope in the STO/LTO interface functions as a repulsive potential giving rise to a small shift in *d*_xy_ density in the LTO region. Additionally, the maximum value of Ti-*d* occupancy in the BTO region is ~0.2, which exceeds the critical concentration for ferroelectric instability (0.11 per 5 atom unit cell) in bulk BTO^15^. This interesting observation implies that the charge inhomogeneity and orbital polarization may stabilize the electric polarization, which requires further theoretical investigation. We note that there are sizable differences in the relative magnitude of the charge density in the LTO and BTO region between the theory and experiment. Since the charge transfer from LTO to STO/BTO region depends mainly on the band alignments between Ti-*d* bands of LTO and STO/BTO^24,25^, the difference may come from the error in the band alignment in the GGA + *U* scheme (see the discussion in the Methods). Another reason for the differences may come from the direct comparison between the integrated local density of Ti*-d*-derived states and the Ti valence obtained from EELS. We perform the Bader charge analysis^26^ to check the dependence in calculational methods and find that the difference in the charge around Ti atoms between LTO and BTO region is reduced about four times due to the re-hybridization effect^27^, but the characteristic feature of the density profile is maintained for both methods, showing the consistency with respect to the methods used in the comparison.

To summarize, the observed periodic modulation of electric polarization, charge distribution, and orbital occupation are schematically shown in Fig. 4c. As seen, the polar structure coexists with a 2DEG forming an artificial 2D polar metal at room temperature with the orbital polarization (i.e., in-plane *d*_xy_ state) controlled by crystal field engineering. Since coexisting 2D superconductivity and magnetism have been recently reported in electron-doped SrTiO_3_^28–32^, we can conjecture that such an artificial non-centrosymmetric 2D metal may provide a mean to engineer an interesting quantum many-body state with three coexisting phases—ferroelectricity, ferromagnetism, and superconductivity^33^.

## Methods

### Sample synthesis and characterization

All films studied in this work were grown on (110)-oriented (orthorhombic notation, corresponding to (001)-orientation in pseudocubic notation) TbScO_3_ substrates (5 × 5 × 0.5 mm^3^) by pulse laser deposition, using a KrF excimer laser operating at *λ* = 248 nm and 2 Hz pulse rate with 2 J/cm^2^ fluence. The layer-by-layer growth was monitored by in situ RHEED. During the growth, the oxygen pressure was kept at ~10^−6^ Torr, whereas the temperature of the substrates was ~850 °C (from reader of infrared pyrometers). As one of the three components in tri-color superlattice (BaTiO_3_)_10_/(SrTiO_3_)_3_/(LaTiO_3_)_3_, bulk BaTi^4+^ O_3_ with 3*d*^0^ electron configuration, a well-known ferroelectric material undergoes complex structural and ferroelectric phase transitions on cooling, e.g., from cubic to tetragonal near 400 K, tetragonal to orthorhombic near 280 K, and orthorhombic to rhombohedral near 210 K (ferroelectric properties are present in the later three phases)^7,34,35^; bulk SrTi^4+^ O_3_ with 3*d*^0^ electron configuration is a band insulator (gap size ~3.3 eV)^36^; bulk LaTi^3+^ O_3_ with 3*d*^1^ electron configuration is a Mott insulator and undergoes G-type antiferromagnetic transition <146 K^37^. In bulk, the lattice parameters are *a* = 3.905 Å for cubic SrTiO_3_; *a* = 4.00 Å for cubic BaTiO_3_; *a* = 5.466, *b* = 5.727, *c* = 7.915, $\sqrt {a^2 + b^2}$/2 = 3.9584, *c*/2 = 3.9575 Å for orthorhombic TbScO_3_; *a* = 5.595, *b* = 5.604, *c* = 7.906, $\sqrt {a^2 + b^2}$/2 = 3.959, *c*/2 = 3.953 Å for orthorhombic LaTiO_3_. Therefore, the SrTiO_3_ layers of BaTiO_3_/SrTiO_3_/LaTiO_3_ superlattice on TbScO_3_ substrate are under tensile strain, whereas the BaTiO_3_ layers are under compressive strain. To guarantee the intriguing electronic states arise from the interfaces of (BaTiO_3_)_10_/(SrTiO_3_)_3_/(LaTiO_3_)_3_ and are not induced by the BaTiO_3_, SrTiO_3_, and LaTiO_3_ layers themselves due to the oxygen doping (LaTiO_3+*δ*_) or vacancies (SrTiO_3−*δ*_ and BaTiO_3−*δ*_), the right Ti valence states in single BaTiO_3_, SrTiO_3_, and LaTiO_3_ films were confirmed by X-ray absorption spectroscopy (XAS) and temperature-dependent electrical transport. The structural quality of the superlattices is further confirmed by X-ray diffraction (XRD) using Cu K_*α*1_ radiation. Electrical transport properties of all films were measured in Van der Pauw geometry by a physical properties measurement system (PPMS, quantum design) operating in high-resolution d.c. mode. The sheet carrier density (*n*_s_) is estimated from *n*_s_ = 1/(*eR*_H_), where the Hall coefficient *R*_H_ is calculated with *R*_H_ = *V*_H_/*IB* (*V*_H_ is the Hall voltage, *I* is the driven current, and B is the out-of-plane magnetic field). As seen in Fig. 1c, there are four metallic STO/LTO interfaces in the superlattice BTO/STO/LTO. Therefore, the carrier density per interface is *n* = *n*_s_/4.

### STEM/EELS measurement

Cross-sectional TEM samples are prepared by focused ion beam with Ga^+^ ions followed with gentle Ar^+^ ion milling^38^. STEM/EELS experiments are performed on a double aberration-corrected microscope JEOL-ARM200F operating at 200 keV, with a dual energy-loss spectrometer and a cold-emission electron source. The structural features of the films are studied by atomic resolution HAADF- and ABF-STEM imaging. Collection angles for HAADF- and ABF-STEM imaging are 67–75 mrad and 11–23 mrad, respectively, and the convergence semi-angle is about 21 mrad. The atomic positions are obtained using 2D Gaussian fitting of the image intensities . When collecting EELS spectra, the microscope conditions are optimized for EELS acquisition with a probe size of ~0.9 Å and a convergence semi-angle about 20 mrad. Line-scanning EELS spectra across the BTO/STO/LTO interfaces are acquired with a step size of 0.12 Å, and a dwell time of 0.05 s per pixel. The energy resolution is about 0.7 eV with a dispersion of 0.1 eV/channel . To measure the elemental concentration of all the cations, a lower dispersion (1 eV/channel) is selected to simultaneously collect Ti L_2,3_-, O K-, Ba M_4,5_-, La-M_4,5_, Sc L_2,3_-, and Tb M_4,5_-edges. To extract the bonding information, a higher energy dispersion (0.1 eV/channel) is chosen for fine structure analysis of Ti L_2,3_-edge. Dual EELS mode is used to simultaneously acquire both the zero-loss and core-loss EELS spectra to ensure that the intrinsic energy drift during spectrum acquisition can be compensated. The background of EELS spectra is subtracted with a power-law function and multiple scattering was corrected by deconvolution. The EELS elemental profile is obtained by integrating the Ti L_2,3_-, O K-, Ba M_4,5_-, La-M_4,5_, Sc L_2,3_-, and Tb M_4,5_-edges, respectively.

### SHG measurement

SHG measurements are performed in a far-field transmission geometry using an 800 nm fundamental laser beam generated by a Spectra-Physics Solstice Ace Ti:Sapphire femtosecond laser system (<100 fs, 1 kHz). The schematic of experimental setup is shown in Fig. 2a, where a linear polarized fundamental 800 nm beam with polarization direction *φ* is incident on the sample at a tilted angle *θ*, the transmission SHG field is first spectrally filtered, then decomposed into s-/p-polarized components (*E*_2*ω*,s_/*E*_2*ω*,p_) and finally detected by a photon multiplier tube. The in-plane orientation of the sample is controlled by angle *β*. Systematic polarimetry is done by scanning polarization direction *φ* with fixed *θ* and *β*.

Theoretical modeling of SHG data is described below. The fundamental field with polarization direction *φ* inside the sample can be written as

1 $${E}_{\omega ,1}' = \left[ {{\mathrm{cos}}\left( {\theta {\prime}} \right){\mathrm{cos}}(\beta ){\mathrm{cos}}(\varphi )t_{\mathrm{p}} - {\mathrm{sin}}(\beta ){\mathrm{sin}}(\varphi )t_{\mathrm{s}}} \right]E_\omega,$$

2 $$E_{\omega ,2}' = \left[ {{\mathrm{cos}}\left( {\theta {\prime}} \right){\mathrm{sin}}(\beta ){\mathrm{cos}}(\varphi )t_{\mathrm{p}} + {\mathrm{cos}}(\beta ){\mathrm{sin}}(\varphi )t_{\mathrm{s}}} \right]E_\omega,$$

3 $$E_{\omega ,3}' = - {\mathrm{sin}}\left( {\theta {\prime}} \right){\mathrm{cos}}(\varphi )t_{\mathrm{p}}E_\omega,$$

where sin(*θ*′) = sin(*θ*)/*n*, *n* is the refractive index, *t*_p_ = 2 cos(*θ*)/(*n* cos(*θ*) + cos(*θ*′)) and *t*_s_ = 2 cos(*θ*)/(cos(*θ*) + *n*cos(*θ*′)) are Fresnel coefficients, *E*_*ω*_ is the magnitude of electric component in air. Under Voigt notation, the SHG field generated through nonlinear optical process can be expressed as $E_{2\omega ,i}' = d_{ij}E_{\omega ,j}'{ {\mathrm{Voigt}}}$, where normalized SHG **d** matrix for *4mm* and *mm*2 symmetry is

4 $${\bf{d}}^{4mm} = \left( {\begin{array}{*{20}{c}} 0 & 0 & 0 & { 0} & {d_{15}} & 0 \\ 0 & 0 & 0 & {d_{15}} & 0 & 0 \\ {d_{31}} & {d_{31}} & {d_{33}} & 0 & 0 & 0 \end{array}} \right),$$

5 $${\bf{d}}^{mm2} = \left( {\begin{array}{*{20}{c}} 0 & 0 & 0 & 0 & {d_{15}} & 0 \\ 0 & 0 & 0 & {d_{24}} & 0 & 0 \\ {d_{31}} & {d_{32}} & {d_{33}} & 0 & 0 & 0 \end{array}} \right).$$

In practice, all the coefficients in **d** tensor are normalized to *d*_15_. By ignoring the index dispersion, that is, *n* = *n*_*ω*_ ≈ *n*_2*ω*_. The transmitted SHG field is

6 $$E_{2\omega ,{\mathrm{p}}} = \left[ {{\mathrm{cos}}\left( {\theta {\prime}} \right){\mathrm{cos}}(\beta ){E}_{2\omega ,1}' + {\mathrm{cos}}\left( {\theta {\prime}} \right){\mathrm{sin}}(\beta )E_{2\omega ,2}' - {\mathrm{sin}}\left( {\theta {\prime}} \right)E_{2\omega ,3}' } \right]t_{\mathrm{p}}',$$

7 $$E_{2\omega ,{\mathrm{s}}} = \left[ { - {\mathrm{sin}}(\beta )E_{2\omega ,1}' + {\mathrm{cos}}(\beta )E_{2\omega ,2}' } \right]t_{\mathrm{s}}',$$

where $t_{\mathrm{p}}'$ = 2*n* cos(*θ*′)/(*n* cos(*θ*) + cos(*θ*′)), $t_{\mathrm{s}}'$ = 2*n* cos(*θ*′)/(cos(*θ*) + *n* cos(*θ*′)), the subscripts s/p denote s/p components of corresponding field variables. Finally, we have below equations for SHG measured intensity:

8 $$I_{2\omega ,{\mathrm{s}}} = \alpha \left| {E_{2\omega ,{\mathrm{s}}}} \right|^2,$$

9 $$I_{2\omega ,{\mathrm{p}}} = \alpha \left| {E_{2\omega ,{\mathrm{p}}}} \right|^2,$$

where *α* is scaling factor.

### First-principles calculations

We performed first-principles density-functional theory calculations with the generalized gradient approximation plus *U* (GGA + *U*) method using the Vienna ab initio simulation package^39,40^. The Perdew–Becke–Erzenhof parametrization^41^ for the exchange-correlation functional and the rotationally invariant form of the on-site Coulomb interaction^42^ are used with *U* = 3 and *J* = 0.68 eV for the titanium *d* states^43–45^ and *U*_*f*_ = 11 and *J*_*f*_ = 0.68 eV for the lanthanum *f* bands away from the Fermi energy^46^. Our choice of *U* value reproduces the correct ground states of bulk LaTiO_3_ and LaTiO_3_/SrTiO_3_ and LaAlO_3_/SrTiO_3_ superlattices^43,45,47^ while maintaining reasonable agreement in the bulk band alignments between filled Ti-*d*-derived lower Hubbard band of LaTiO_3_ and empty Ti-*d*-derived states of SrTiO_3_ and BaTiO_3_ compared with those from the hybrid functional^48–50^. The projector augmented-wave method^51^ are used with pseudopotentials contain six valence electrons for O (2*s*^2^2*p*^4^), 12 for Ti (3*s*^2^3*p*^6^3*d*^2^4*s*^2^), 10 for Sr (4*s*^2^4*p*^6^5*s*^2^), 10 for Ba (5*s*^2^5*p*^6^6*s*^2^), and 11 for La (5*s*^2^5*p*^6^5*d*^1^6*s*^2^). We used an energy cutoff of 500 eV and *k*-point sampling on a 8 × 8 × 1 grid with 1 × 1 in-plane unit cell. Although our choice of the unit cell does not include the GdFeO_3_ type of octahedral rotation and tilts observed in bulk LaTiO_3_^52,53^, we find that the orbital polarization of Ti-*d* states for (LaTiO_3_)_2_/(SrTiO_3_)_4_ superlattices are similar in the magnitude regardless of the presence of the octahedral rotation and tilts for the interface and SrTiO_3_ region with some deviations in the LaTiO_3_ region. Since our main focus is the properties of the interface electron gas, it is reasonable to use of the 1 × 1 in-plane unit for the large unit-cell size of the tri-color superlattices. The atomic positions and *c*-lattice constants are fully relaxed with 0.02 eV per Å force threshold while *a* and *b* lattice constants are fixed to experimental in-plane lattice constants of TbScO_3_. The layer-resolved charge distribution in Fig. 4b and Supplementary Fig. 5a, b are calculated by integrating the Ti-*d*-projected density of states in the energy windows from −1 eV to the Fermi energy (Supplementary Fig. 5c) and is normalized to satisfy that the total number of electron is the same as the number of LaO layers.

### Data availability

The data and code that support the findings of this study are available from the corresponding authors upon reasonable request.

## Electronic supplementary material

Supplementary Information
