# Supplementary material for: Artificial two-dimensional polar metal at room temperature
Source: Nat Commun. 2018 Apr 18;9:1547. doi: 10.1038/s41467-018-03964-9 (PMC5906683; doi:10.1038/s41467-018-03964-9)
Supplement: Supplementary file 1 — Supplementary Information [file 41467_2018_3964_MOESM1_ESM.pdf]

Artificial two-dimensional polar metal at room temperature **Cao et al.**

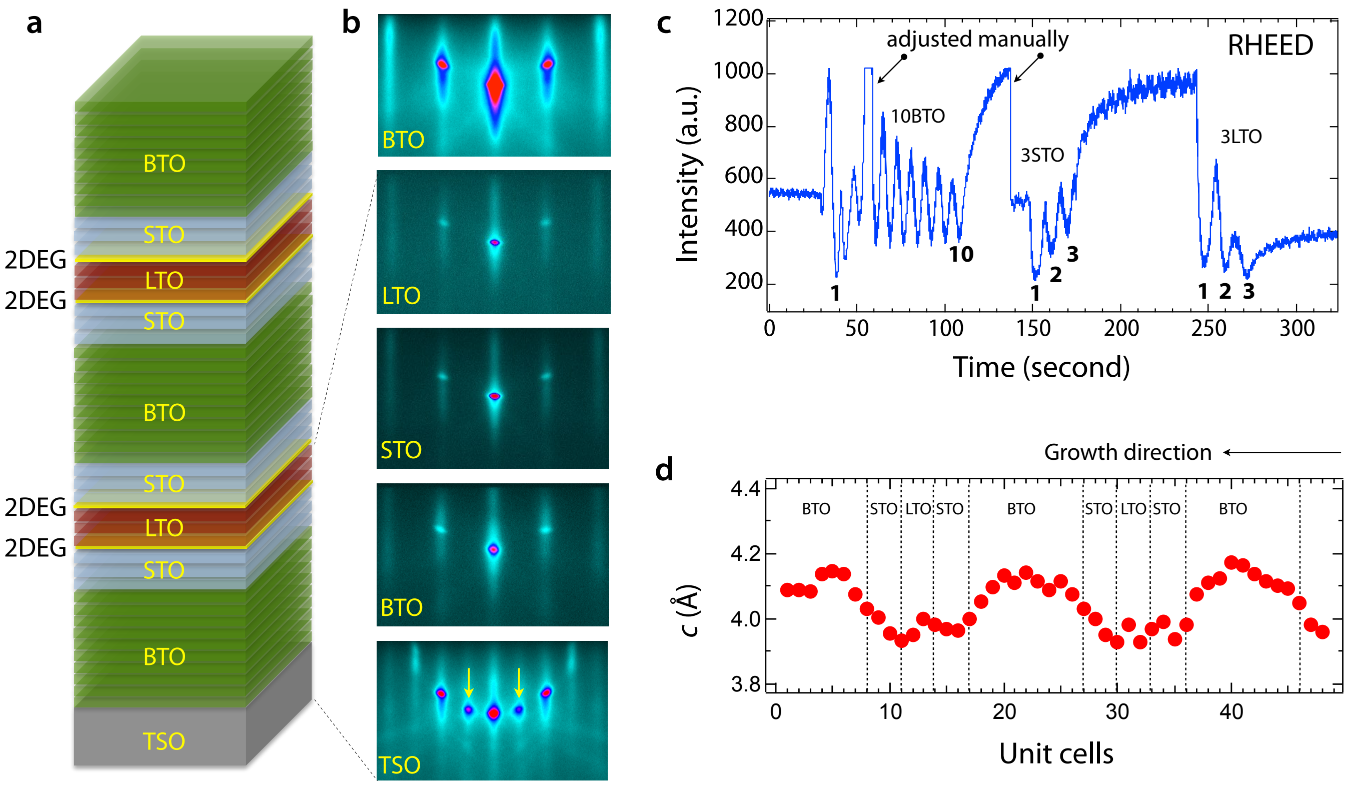

Supplementary Figure 1 Sample synthesis and characterization. **a** Block sketch of growth sequences of $BaTiO_3/SrTiO_3/LaTiO_3$ superlattice, in which two-dimensional electron gases (2DEG) are formed at $SrTiO_3/LaTiO_3$ interfaces. **b** RHEED patterns for $TbScO_3$ (TSO) substrate, $BaTiO_3$ (BTO), $SrTiO_3$ (STO), $LaTiO_3$ (LTO), and the top $BaTiO_3$ (BTO) layers, respectively, during the growth of $(BTO)_{10}/(STO)_3/(STO)_3$ (where the subscript refers to the number of unit cells). Yellow arrows indicate half-order-peaks of the orthorhombic structure of TSO substrate. **c** Partial RHEED intensity oscillation during growth of $(BTO)_{10}/(STO)_3/(STO)_3$. **d** Layer-dependent out-of-plane lattice parameter *c* measured from STEM image, demonstrating the periodic synthesis of BTO, STO, and LTO layers of $(BTO)_{10}/(STO)_3/(STO)_3$.

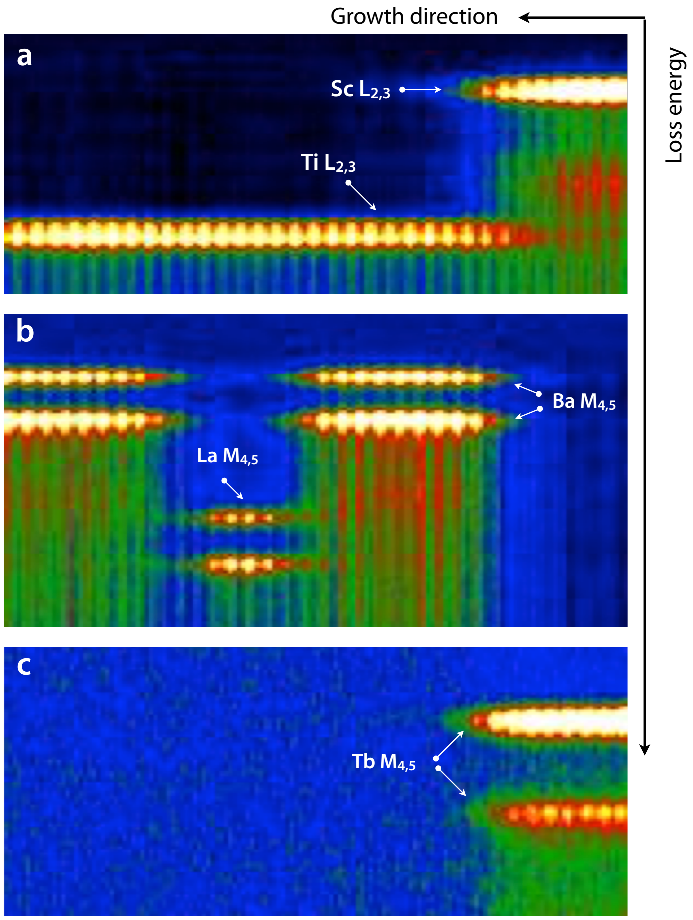

Supplementary Figure 2 EELS spectra maps of 10BTO/3STO/3LTO. **a** Ti $L_{2,3}$- and Sc $L_{2,3}$-edges spectra. **b** Ba $M_{4,5}$- and La $M_{4,5}$-edges. **c** Tb $M_{4,5}$-edge.

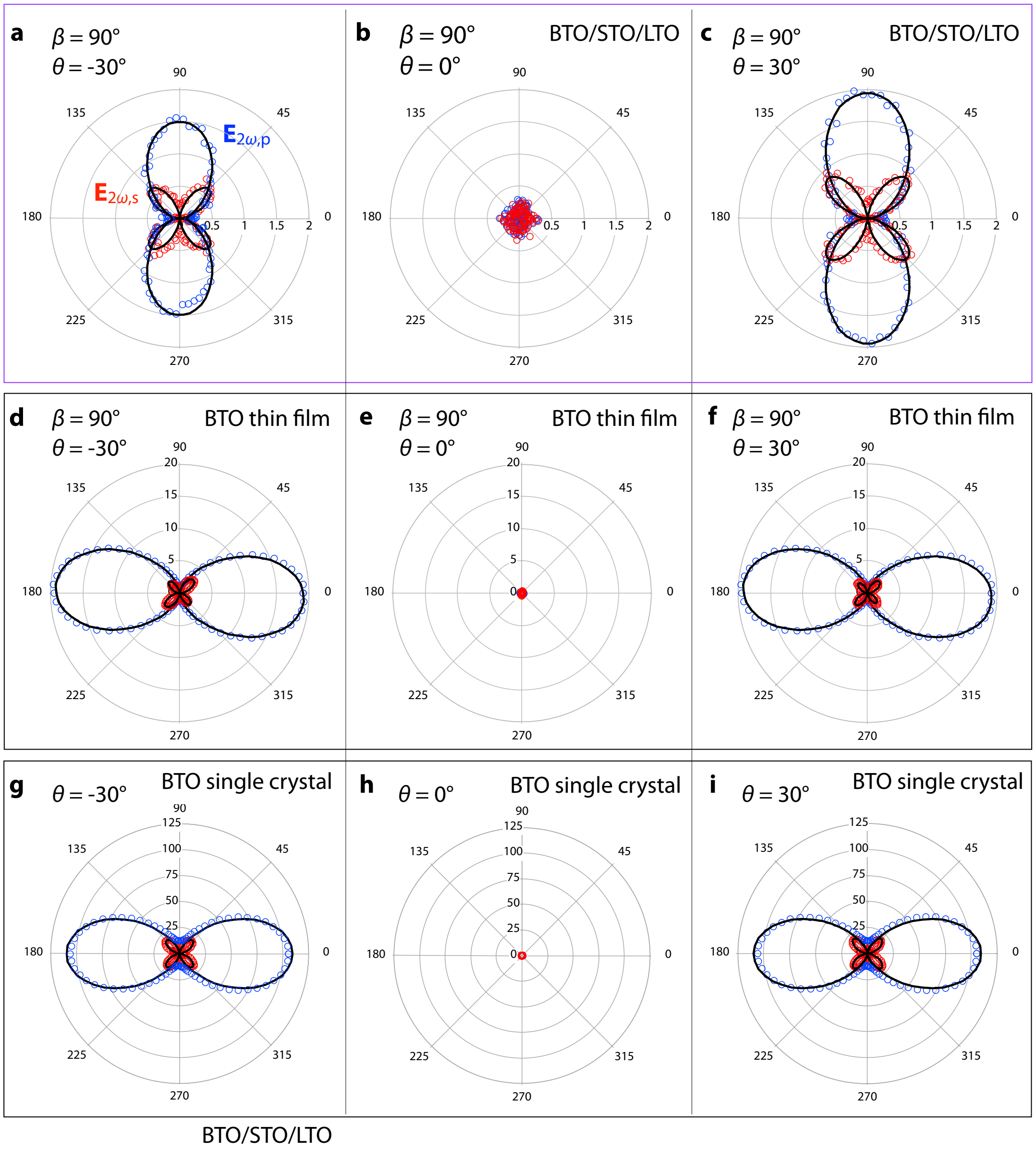

Supplementary Figure 3 SHG polarimetry measurement. $E_{2\omega,\mathrm{p}}$ (blue circles) and $E_{2\omega,\mathrm{s}}$ (red circles) SHG signal on **a-c** 10BTO/3STO/3LTO, **d-f** 10BTO thin film, and **g-i** BTO single crystal measured by rotating the incident polarization φ under three different incident angles θ = -30°, 0°, 30°. Data with β = 90° are plotted here. Theoretical modeling of SHG signal (black lines) indicates $4mm$ point group symmetry for 10BTO/3STO/3LTO with effective nonlinear optical coefficients $d_{33}/d_{15} \approx$ -13.9, $d_{31}/d_{15} \approx$ 1.3. In comparison, SHG signal on 10BTO thin film and BTO single crystal exhibit $mm2$ point group symmetry with $d_{33}/d_{15} \approx$ 5.2, $d_{31}/d_{15} \approx$ 0.1 and $d_{33}/d_{15} \approx$ 3.3, $d_{31}/d_{15} \approx$ 0.3, respectively.

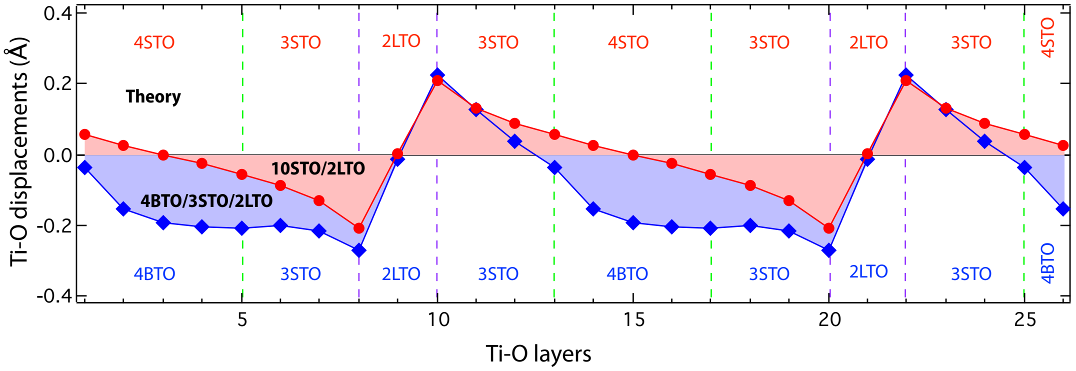

Supplementary Figure 4 Calculated Ti-O polar displacements. The comparison of polar displacements between tri-color 4BTO/3STO/2LTO (blue diamonds) and reference sample 10STO/2LTO (red dots) indicates the LTO layers plays an important role for the reversal of Ti-O polar displacements whereas the net polarization in 4BTO/3STO/2LTO is driven by polar distortions in BTO layers.

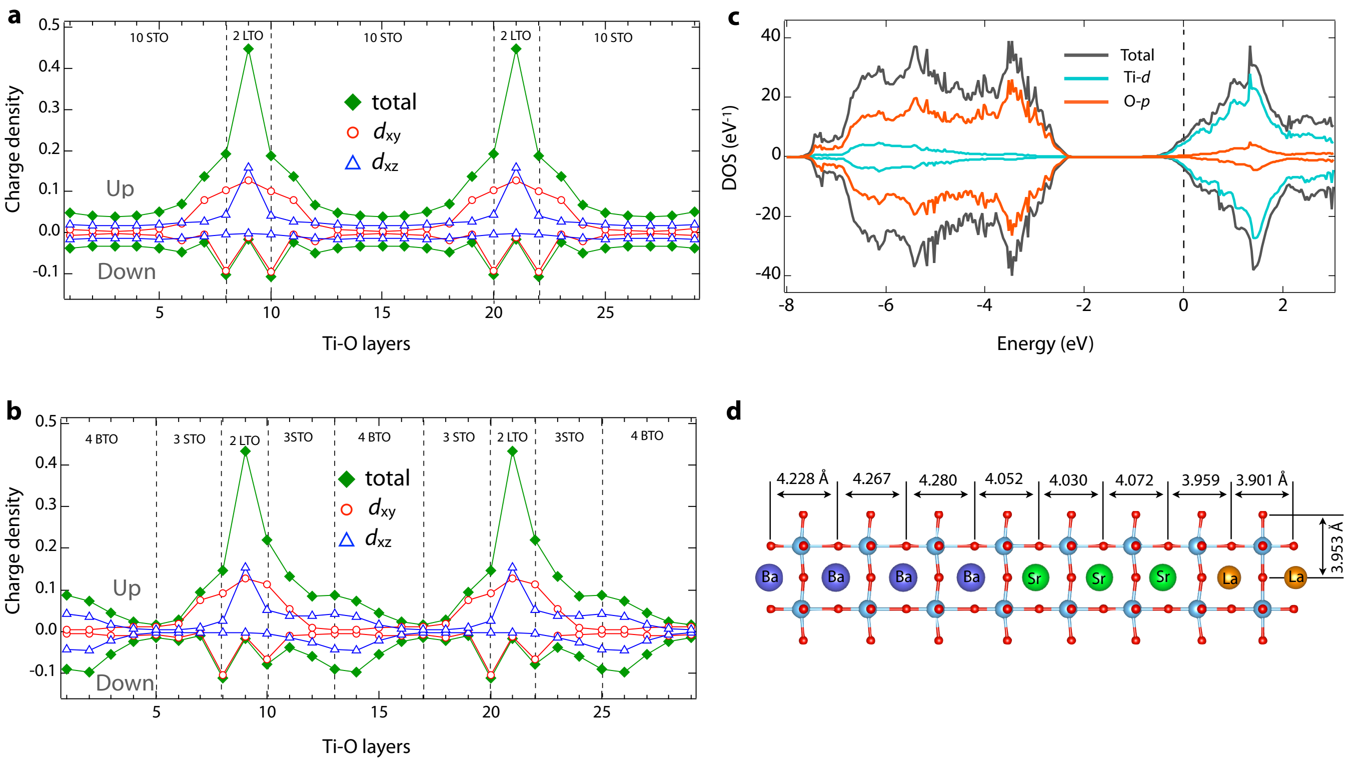

Supplementary Figure 5 Calculated orbitally resolved charge distribution for each TiO_2 layer. **a** 10STO/2LTO. **b** 4BTO/3STO/2LTO. **c** Orbitally resolved projected density of state of 4BTO/3STO/2LTO superlattice. **d** Calculated lattice parameters of TiO_6 octahedra across 4BTO/3STO/2LTO interfaces.
